# Serum Levels of Interleukin-9 Correlate with Negative Prognostic Factors in Extranodal NK/T-Cell Lymphoma

**DOI:** 10.1371/journal.pone.0094637

**Published:** 2014-04-10

**Authors:** Jing Zhang, Wei-da Wang, Qi-rong Geng, Liang Wang, Xiao-qin Chen, Cheng-cheng Liu, Yue Lv

**Affiliations:** 1 Department of Hematologic Oncology, Sun Yat-Sen University Cancer Center, Guangzhou, Guangdong, P. R. China; 2 State Key Laboratory of Oncology in South China, Guangzhou, Guangdong, P. R. China; 3 Department of Medical Oncology, Hubei Cancer Hospital, Wuhan, Hubei, P. R. China; National Cancer Center, Japan

## Abstract

Interleukin-9 (IL-9) is more functionally diverse than previously expected, especially with regards to lymphomagenesis. However, the relationship between IL-9 and the clinicopathological features of extranodal NK/T-cell lymphoma is less well established. Patients with this lymphoma in Sun Yat-Sen University Cancer Center between January 2003 and March 2013 were systematically reviewed in an intention-to-treat analysis. Baseline serum IL-9 levels were determined using sandwich enzyme-linked immunosorbent assays. A total of seventy-four patients were enrolled in this study. The mean concentration of serum IL-9 for all patients was 6.48 pg/mL (range: 1.38–51.87 pg/mL). Age, B symptoms and local lymph node involvement were found to be related to high serum IL-9 levels. Patients with low IL-9 levels tended to have higher rates of complete remission. Notably, the median progression-free survival (PFS) and overall survival (OS) were longer in the low IL-9 level group than in the high IL-9 level group (PFS: 68.7 months vs. 28.3 months, *P*<0.001; OS: 86 months vs. 42.8 months, *P* = 0.001). Multivariate analysis revealed independent prognostic factors for PFS. Similarly, high IL-9 levels (*P* = 0.003) and old age (*P* = 0.007) were independently predictive of shorter OS. Serum IL-9 is closely related to several clinical features, such as age, B symptoms and local lymph node involvement. It can also be a significant independent prognostic factor for extranodal NK/T-cell lymphoma, which suggests a role for IL-9 in the pathogenesis of this disease and offers new insight into potential therapeutic strategies.

## Introduction

Extranodal NK/T-cell lymphoma (ENKTL) is more prevalent in Asia than in Western countries [1], [2]. According to a recent large survey [3], it is the second most common lymphoma subtype in China and is only inferior to diffuse large B-cell lymphoma. It is characterized by vascular damage, zonal tumor cell death and prominent tissue necrosis. Many studies have suggested the involvement of cytokines in these pathological changes, such as CXCL9, CXCL10 [4], [5], and interleukin-9 (IL-9) [6].

IL-9 has attracted renewed interest since its expression was identified in multiple T helper (Th) cell subsets (including Th2, Th9, Th17), regulatory T cells (Treg) and natural killer (NK)/T cells [7]. Several studies have indicated that IL-9 may promote oncogenesis during Hodgkin's Lymphoma (HL) and large cell anaplastic lymphoma [8]–[10] in addition to its significant regulatory role in allergy and autoimmunity [11]–[13]. Dysregulation of the Janus kinase (JAK)/signal transducer and activator of transcription (STAT) pathway may be responsible for the oncogenesis of IL-9 *in vitro* and *in vivo* [14]–[16].

Correlations have been found between IL-9 levels and negative prognostic factors, including advanced stage, B-symptoms (including weight loss >10%, fever, drenching night sweats), low blood hemoglobin and elevated erythrocyte sedimentation rates in HL [8]. Nagato T *et al.* also reported that IL-9 was present in biopsy samples and serum from patients with ENKTL and played an important role in the disease, possibly via an autocrine mechanism [6]. These results suggest that IL-9 may be associated with the clinicopathological features of ENKTL, although little is known about the relationship between them. To clarify this problem, we measured IL-9 levels in the serum of patients with ENKTL and explored the clinical significance of serum IL-9 during ENKTL.

## Methods

### Ethics statement

This study was approved by the Institutional Review Board of Sun Yat-Sen University Cancer Center, and written informed consent was obtained from all healthy volunteers and patients prior to treatment. Additionally, this study was conducted in accordance with the Helsinki Declaration.

### Eligibility criteria

This study used a retrospective cohort study design. Patients with nasal ENKTL who received chemotherapy, radiotherapy or both in Sun Yat-Sen University Cancer Center between January 2003 and March 2013 were systematically reviewed in an intention-to-treat analysis. All eligible cases were selected consecutively. Eligibility criteria for inclusion in this study were as follows: (1) pathologically confirmed diagnosis of ENKTL according to the World Health Organization classification; (2) positive for CD3, CD56, cytotoxic molecules, and Epstein-Barr virus by in situ hybridization and negative for CD20; (3) primary symptoms and the bulk of the tumor localized to the nasal cavity; (4) no previous anti-tumor treatments; (5) available serum samples obtained before the primary treatment and stored at −80°C; (6) complete follow-up results. The exclusion criteria were: (1) prior or concomitant malignant tumors; (2) any co-existing medical problems of sufficient severity to prevent full compliance with standard antitumor therapy protocols; (3) other subtypes of non-Hodgkin lymphoma (NHL), including myeloid/NK cell precursor acute leukemia, blastic NK cell lymphoma/precursor NK cell lymphoblastic leukemia, aggressive NK cell leukemia, and peripheral T cell lymphoma.

All enrolled patients underwent standard Ann Arbor staging with history, physical examination, nasopharyngeal endoscopy, whole body positron emission tomography/computed tomography (PET/CT) scans, CT scans or magnetic resonance imaging of the involved organs of the head and neck and CT scans of the chest, abdomen and pelvis. Complete blood counts and serum biochemistry were routinely examined.

### Treatment

Patients received one of the following initial treatment modalities: (1) chemotherapy followed by involved-field radiotherapy; (2) chemotherapy alone; (3) radiotherapy alone. The following chemotherapy regimens were included: (1) CHOP (cyclophosphamide, doxorubicin, vincristine, prednisone); (2) EPOCH (etoposide, doxorubicin, vincristine, cyclophosphamide, prednisone); (3) alternating triple therapy (ATT): CHOP-B (CHOP plus bleomycin), IMVP-16 (ifosfamide, methotrexate, etoposide), DHAP (dexamethasone, cisplatin, cytarabine); (4) GELOX (gemcitabine, oxaliplatin, L-asparaginase) or modified GELOX [17]; (5) others: CHOP-L (CHOP plus L-asparaginase), DeVIC (dexamethasone, etoposide, ifosfamide, carboplatin). Patients received at least one cycle and a maximum of eight cycles of initial chemotherapy. Involved-field radiotherapy of 50–60 Gy was delivered in daily fractions of 1.8–2.0 Gy with five fractions each week. The treatment response was assessed according to the standardized response criteria for non-Hodgkin lymphomas [18]. After the completion of treatment, patients were followed by their ambulatory oncologists. The follow-up interval was based on the regular standard. Overall survival (OS) was measured from the time of diagnosis until death from any cause. Progression-free survival (PFS) was measured from the time of diagnosis until disease progression, relapse, or death from any cause or until the last follow-up.

### ELISA

Serum IL-9 levels were determined using sandwich enzyme-linked immunosorbent assay (ELISA) kits (Human IL-9 Platinum ELISA; Bender MedSystems, Vienna, Austria). All venous blood samples were drawn from ten healthy volunteers and from patients at diagnosis. The samples were centrifuged at 4°C, and serum was collected and quickly frozen at −80°C until further assay. A routine ELISA method was performed according to the manufacturer's protocol. The limit of detection of human IL-9 was 0.5 pg/mL. All samples were analyzed in duplicate, experiments were repeated three times, and the results are presented as the means ± standard deviations.

### Statistical analysis

Differences in the mean values were tested using the non-parametric Mann-Whitney U-test. The cut off concentration of serum IL-9 for survival analysis was determined using the receiver operating characteristics (ROC) curve analysis. The correlation between serum IL-9 levels and complete remission (CR) rates was performed using the Chi-square test. Univariate analyses of the effects of several pretreatment characteristics upon survival, including IL-9, were performed using the Kaplan-Meier method and log-rank test. Multivariate analysis was performed using the Cox proportional-hazards regression technique to define the prognostic significance of selected variables, including IL-9. A two-sided *P* value <0.05 was considered statistically significant. All statistical analyses were performed using PASW Statistics 18.0 software (Apache Software Foundation, Forest Hill, MD).

## Results

### Patient characteristics and serum IL-9 content at baseline

A total of seventy-four patients were enrolled in this study. The clinical characteristics are listed in Table 1. The ratio of males to females was 3.11∶1, with a median age of 42.5 years. Seventy-one patients (95.9%) had a good performance status (Eastern Cooperative Oncology Group 0–1), and B symptoms occurred in more than half of the patients. Sixty-two patients (83.7%) had stage I-II disease, and the remaining patients had stage III-IV disease. Most patients were assigned to the low-risk group with an International Prognostic Index (IPI) score of 0-1. However, nearly half the patients were grouped in the high-risk group according to the Korean Prognostic Index (KPI) score.

**Table 1:** Clinical characteristics at baseline.

Characteristics	No.	%
Gender, male	56	75.7
Age, y		
Median (range)	42.5 (13–76)	
≤60	63	85.1
ECOG performance status		
0	28	37.8
1–2	46	62.2
B symptoms	39	52.7
Serum LDH (Elevated)	23	31.1
LN involvement	39	52.7
Ann Arbor stage		
I	32	43.2
II	30	40.5
III–IV	12	16.3
IPI		
0–1	61	82.4
2–5	13	17.6
KPI		
0	15	20.3
1	27	36.5
2	16	21.6
3–4	16	21.6
Lymphocytes (<normal)	23	31.1
Monocytes (>normal)	41	55.4
Pretreatment serum IL-9		
Mean concentration, pg/mL (range)	6.48 (1.38–51.87)	

Abbreviations: ECOG, Eastern Cooperative Oncology Group; LDH, lactate dehydrogenase;

LN, local lymph node; IPI, international prognostic index; KPI, Korean prognostic index.

**Figure 1:**
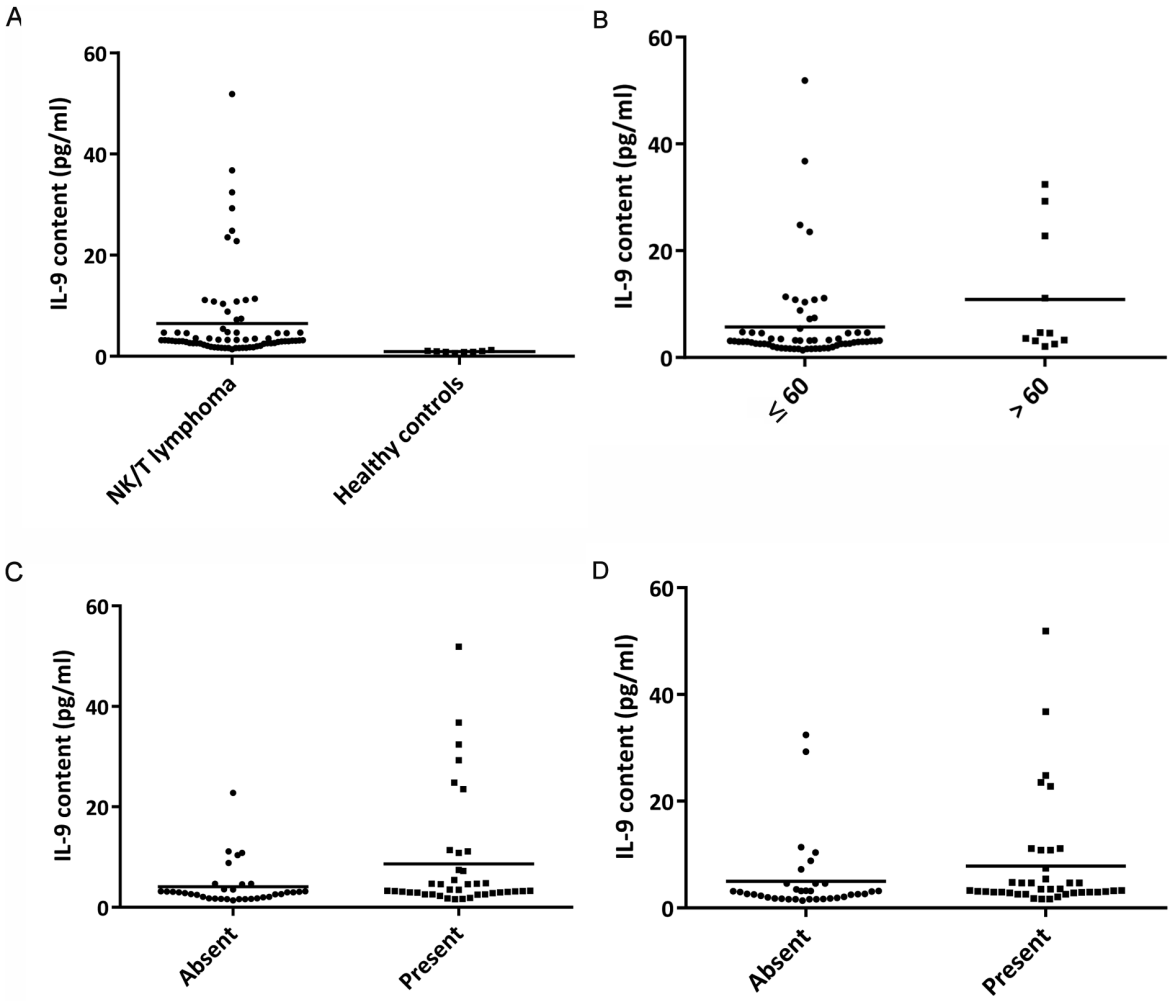
Correlation between serum IL-9 levels and clinical features. A: Serum IL-9 levels were significantly higher in patients with ENKTL than in healthy volunteers (*P*<0.001). B: Serum IL-9 levels in patients >60 years were significantly higher than in those ≤60 years (*P* = 0.042). C: Serum IL-9 levels in patients with B symptoms were significantly higher than in those without B symptoms (*P* = 0.009). D: Serum IL-9 levels in patients with local lymph node involvement were significantly higher than in those without local lymph node involvement (*P* = 0.011).

The mean concentration of serum IL-9 for all patients was 6.48 pg/mL (range: 1.38–51.87 pg/mL) with a median of 3.12 pg/mL. Serum IL-9 was detected in six of ten healthy volunteers, and the average concentration was 0.56 pg/mL (range: 0–1.23 pg/mL), which was significantly lower than that of ENKTL patients (*P*<0.001, Fig. 1A).

### Correlation between serum IL-9 levels and clinical features

To explore the correlation between serum IL-9 levels and prognostic factors, the IL-9 content at baseline was compared according to the clinical features of all patients (Table 2). The IL-9 levels for patients >60 years, with B symptoms and with local lymph node involvement were significantly higher than for those ≤60 years (*P* = 0.042), without B symptoms (*P* = 0.009), or without local lymph node involvement (*P* = 0.011, Fig. 1B–D). Other clinical factors, such as male (*P* = 0.316), poor performance status (*P* = 0.125), elevated LDH (*P* = 0.995), advanced stages (*P* = 0.084), high IPI (*P* = 0.125) or KPI score (*P* = 0.284), low lymphocyte counts (*P* = 0.250) and high monocyte counts (*P* = 0.724), were not significantly associated with high serum IL-9 concentrations.

**Table 2:** The differences of serum IL-9 levels in various clinical factors.

Factors	No.	Average	Range	*P* value
Gender				
Male	56	7.02	1.38–51.87	0.316
Female	18	4.78	1.62–23.52	
Age				
≤60	63	5.71	1.38–51.87	0.042
>60	11	10.85	2.08–32.4	
ECOG performance status				
0	28	4.61	1.38–22.8	0.125
1–2	46	7.61	1.62–51.87	
B symptoms				
Absent	35	4.1	1.38–22.8	0.009
Present	39	8.61	1.62–51.87	
Serum LDH				
Normal	51	5.61	1.62–32.4	0.995
>normal	23	8.39	1.38–51.87	
LN involvement				
Absent	35	4.98	1.38–32.4	0.011
Present	39	7.82	1.66–51.87	
Ann Arbor stage				
I	32	5.17	1.38–32.4	0.084
II	30	7.76	1.62–51.87	
III–IV	12	6.74	1.69–24.82	
IPI				
0–1	62	6.38	1.38–51.87	0.125
2–5	12	6.99	2.62–24.82	
KPI				
0	15	5.16	1.38–32.4	0.284
1	27	5.74	1.62–29.28	
2	16	4.59	1.66–11.12	
3–4	16	10.84	1.69–51.87	
Lymphocytes				
Normal	51	7.56	1.38–51.87	0.25
<normal	23	4.07	1.62–23.52	
Monocytes				
Normal	33	6.4	1.38–32.4	0.724
>normal	41	6.54	1.62–51.87	

Abbreviations: ECOG, Eastern Cooperative Oncology Group; LDH, lactate dehydrogenase;

LN, local lymph node; IPI, international prognostic index; KPI, Korean prognostic index.

### Cut-off value for serum IL-9

**Figure 2:**
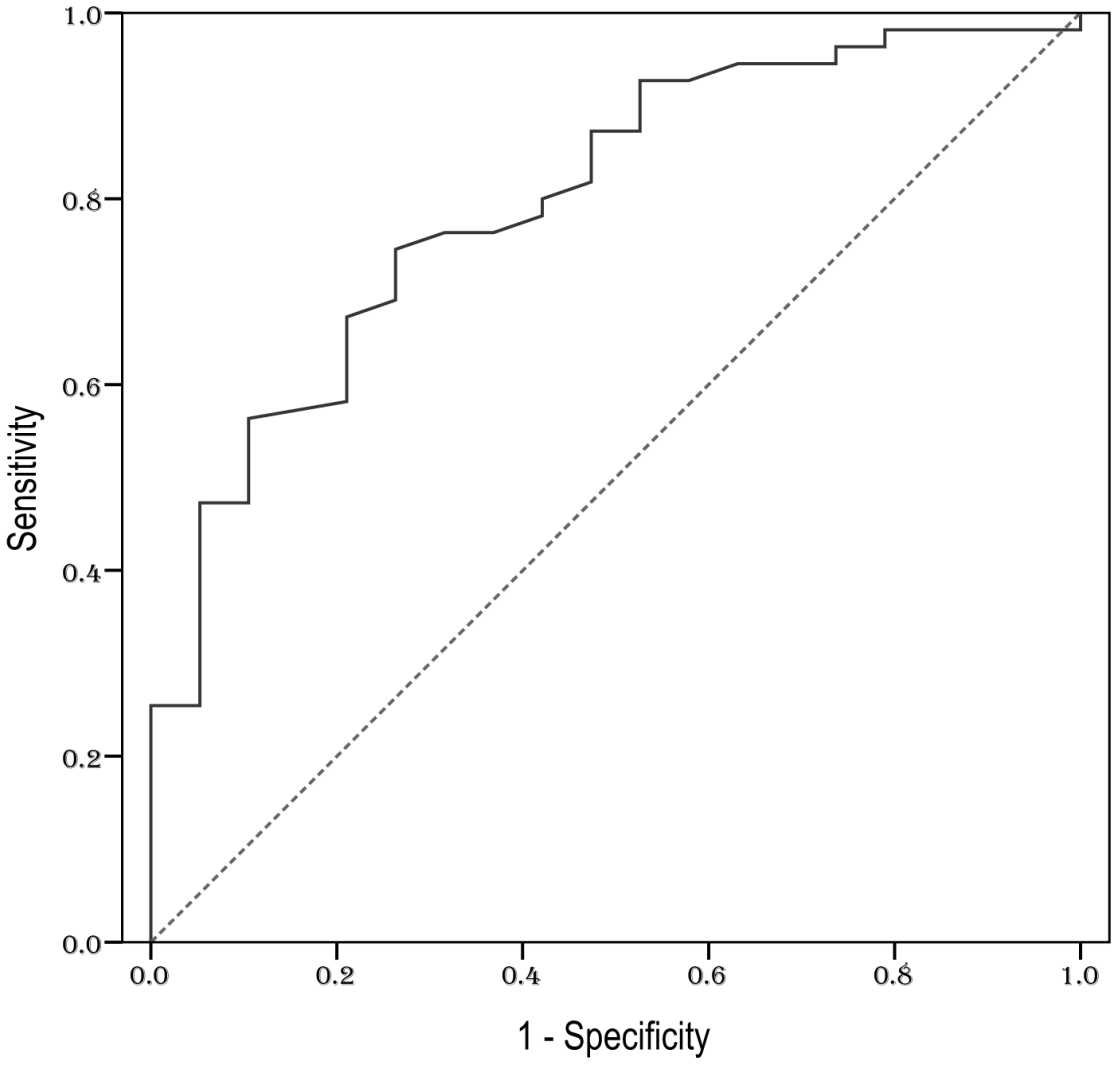
ROC curve analysis for the optimal cut-off point of serum IL-9. The most discriminative cut-off concentration of serum IL-9 was 3.49 pg/mL with an AUC value of 0.799. The sensitivity and specificity were 74.5% and 73.7%, respectively.

To identify an optimal cut-off point for survival outcomes, the ROC curve analysis was selected. The most discriminative cut-off concentration of serum IL-9 was 3.49 pg/mL with an area under the curve (AUC) value of 0.799 [95% confidence interval (CI) 0.688–0.909, *P*<0.001] (Fig. 2). The sensitivity and specificity of the dichotomized IL-9 levels (≤3.49 pg/mL vs. >3.49 pg/mL) were 74.5% and 73.7%, respectively.

### Treatment response and survival

The treatment response was evaluated in each patient: 55 of 74 (74.3%) achieved CR, 8 of 74 (10.8%) achieved partial response, 5 of 74 (6.8%) showed stable disease, and 6 patients (8.1%) showed progressive disease. The CR rates of patients with IL-9 levels ≤3.49 pg/mL and >3.49 pg/mL were 83.3% and 57.7%, respectively (*P* = 0.016).

**Figure 3:**
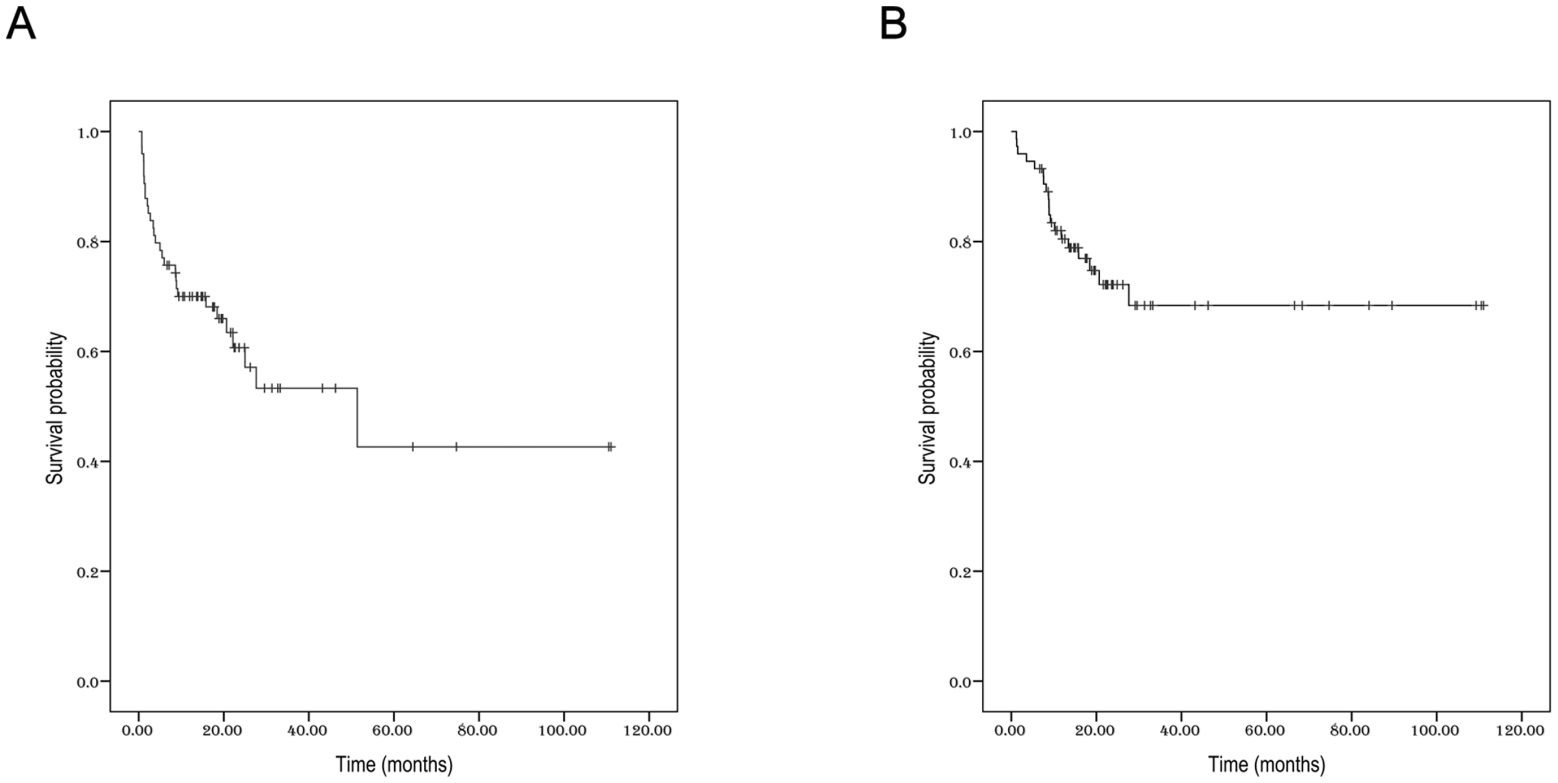
The survival for all patients. A: Progression-free survival. B: Overall survival.

**Figure 4:**
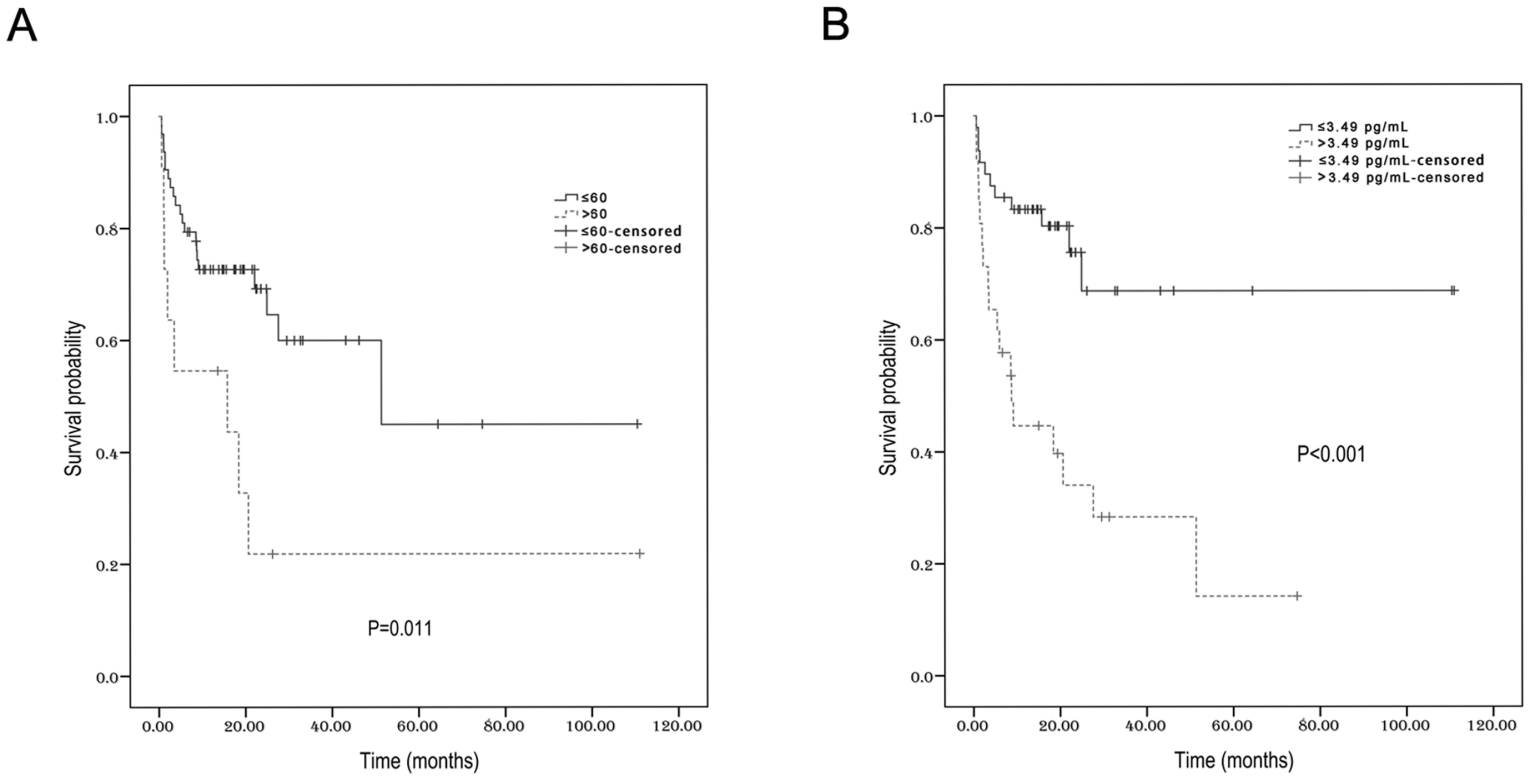
The progression-free survival according to age (A) and serum IL-9 levels (B).

**Figure 5:**
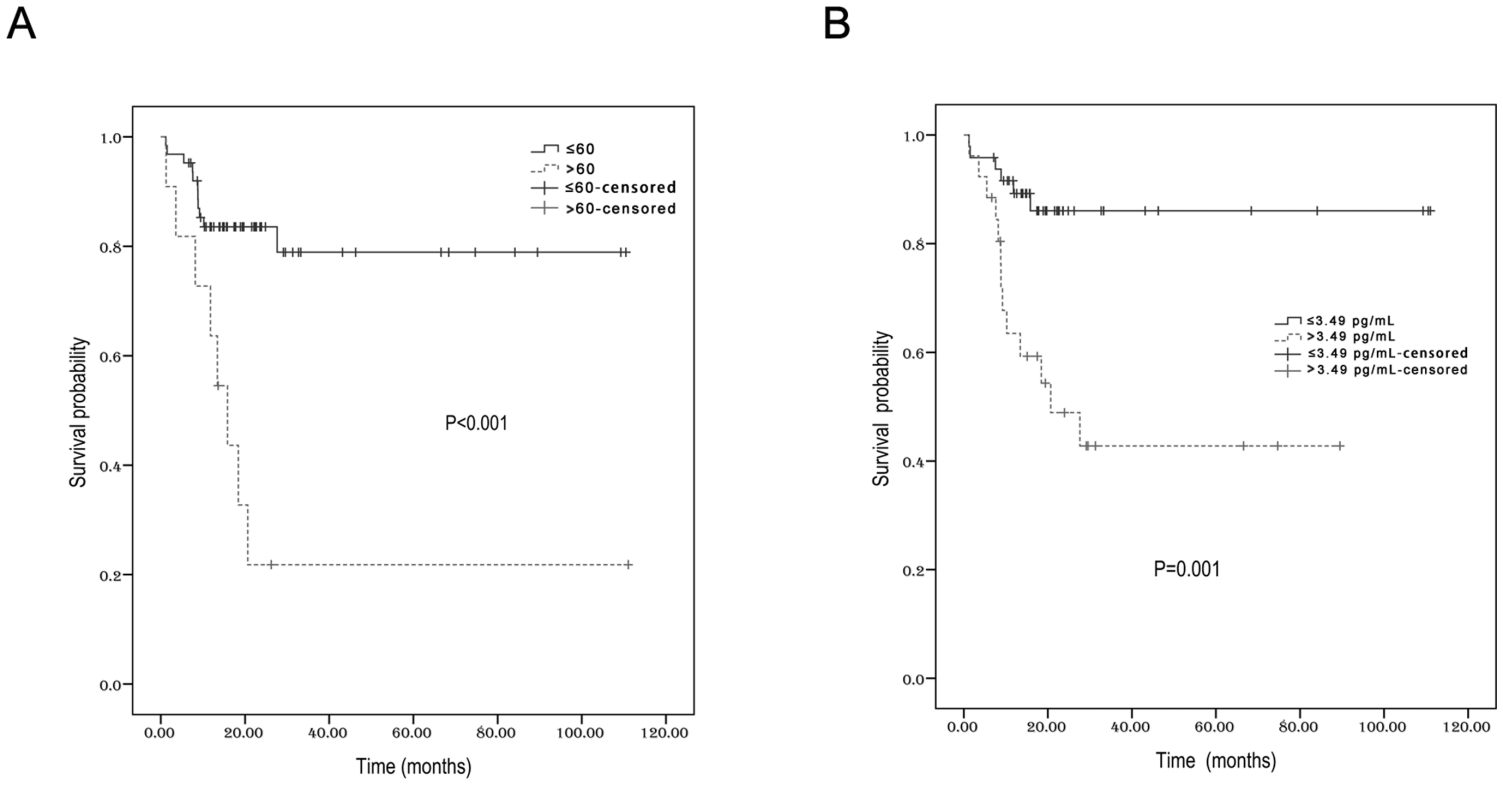
The overall survival according to age (A) and serum IL-9 levels (B).

Within a median follow-up time of 17.7 months, the 3-year PFS and OS for all patients were 53.3% (95% CI 45.8%–60.8%) and 68.4% (95% CI 61.8%–75%), respectively (Fig. 3). Univariate analysis revealed that young age, (≤60 years), no local lymph node involvement, early stage, and low serum IL-9 levels (≤3.49 pg/mL) could significantly predict longer PFS (Table 3, Fig. 4). The 3-year PFS (68.7%) was much higher in patients with low IL-9 content (≤3.49 pg/mL) than in those with high IL-9 content (>3.49 pg/mL) (28.3%, *P*<0.001). In the subgroup with stage I–II disease, low serum IL-9 concentrations were still predictive of better PFS (data not shown). In terms of OS, only age and serum IL-9 levels were predictive for 3-year survival rates. Ann Arbor stage and KPI scores lost their prognostic roles for OS. For patients of no more than 60 years, the 3-year OS was 78.9%, but it was fairly low in patients older than 60 years (21.8%, *P*<0.001, Fig. 5A). In addition, the 3-year OS was as high as 86% for patients with low IL-9 levels. However, the 3-year OS was only 42.8% in those with high IL-9 levels (*P* = 0.001, Fig. 5B).

**Table 3:** The 3-year survival rates according to various clinical factors.

Factors	3-y PFS (%)	95% CI	*P*	3-y OS (%)	95% CI	*P*
Gender			0.117			0.118
Male	45.3	36.4–54.2		61.9	53.7–70.1	.
Female	77.8	68–87.6		88.1	80.2–96	
Age			0.011			<0.001
≤60	60	51.8–68.2		78.9	71.8–85.3	
>60	21.8	8.4–35.2		21.8	8.4–35.2	
ECOG performance status			0.109			0.152
0	60.4	48.5–72.3		76.7	66.8–86.6	
1–2	52	43.6–60.4		63.9	55.7–72.1	
B symptoms			0.122			0.285
Absent	66.8	57.5–76.1		77.9	70.4–85.4	
Present	43	32.8–53.2		61.2	51.5–70.9	
Serum LDH			0.455			0.202
Normal	57.3	48.8–65.8		70.5	62.7–78.3	
>normal	44.7	29.6–59.8		68	58–78	
LN involvement			0.005			0.071
Absent	70.8	61.7–79.9		78.3	70.2–86.4	
Present	35.2	23.7–46.7		58.7	48.3–69.1	
Ann Arbor stage			0.042			0.304
I	68.3	58.6–78		76.2	67.4–85	
II	41.3	27.9–54.7		61.7	50.4–73	
III–IV	41.7	27.5–55.9		66.7	53.1–80.3	
IPI			0.067			0.053
0–1	55.9	47.6–64.2		70.8	63.6–78	
2–5	41.7	27.5–55.9		58.3	44.1–72.5	
KPI			0.064			0.251
0	66	51.9–80.1		73.5	60–87	
1	67.9	58.2–77.6		75.3	66.3–84.3	
2	34.4	9.4–59.4		53.5	30.6–76.4	
3–4	20.1	4.5–35.7		59.1	46.1–72.1	
Lymphocytes			0.286			0.217
Normal	49.3	40.8–57.8		62.5	54.4–70.6	
<normal	62.6	47–78.2		86.7	47.3–93.8	
Monocytes			0.432			0.861
Normal	51.6	41.5–61.7		69	60.1–77.9	
>normal	53.5	42.4–64.6		67.3	57.4–77.2	
Serum IL-9			<0.001			0.001
≤3.49	68.7	59.4–78		86	80.6–91.4	
>3.49	28.3	18.4–38.2		42.8	31.9–53.7	

Abbreviations: PFS, progression-free survival; OS, overall survival; ECOG, Eastern Cooperative Oncology Group; LDH, lactate dehydrogenase; LN, local lymph node; IPI, international prognostic index; KPI, Korean prognostic index

### Multivariate analysis

The clinical factors that were statistically significant predictors of the PFS were included in the multivariate analysis. Multivariate analysis revealed that the independent prognostic factors for PFS were serum IL-9 concentration (*P* = 0.001; relative risk, 3.541; 95% CI 1.626–7.714) and age (*P* = 0.025; relative risk, 1.029; 95% CI 1.004–1.056). Similarly, high IL-9 levels (*P* = 0.003; relative risk, 4.377; 95% CI 1.628–11.766) and old age (*P* = 0.007; relative risk, 1.043; 95% CI 1.012–1.076) were independently predictive of shorter OS (Table 4).

**Table 4:** Multivariate analysis of prognostic factors for survival.

	PFS			OS		
Parameters	*P*	RR	95% CI	*P*	RR	95% CI
Age >60 years	0.025	1.029	1.004–1.056	0.007	1.043	1.012–1.076
LN involvement	0.107	2.650	0.810–8.668	0.386	1.867	0.456–7.654
Serum IL-9 (>3.49 pg/mL)	0.001	3.541	1.626–7.714	0.003	4.377	1.628–11.766
Ann Arbor stage (I vs. II vs. III–IV)	0.945	0.979	0.535–1.791	0.972	1.013	0.496–2.068

Abbreviations: PFS, progression-free survival; OS, overall survival; LN, local lymph node.

## Discussion

IL-9 was first described in the late 1980s as an emerging cytokine with pleiotropic functions in the immune system [19]. It demonstrated pro-inflammatory activity and played a key role in the pathogenesis of atopic diseases and asthma [20]–[22]. Its activities are mediated via a heterodimeric receptor complex formed by the IL-9Rα chain, which associates with JAK1 and the IL-2Rγ chain, also known as γc (common γ chain), which associates with JAK3 [23]. JAK3 mutations are involved in the pathogenesis of NK/T cell lymphoma [24], [25]. With the detection of more immune targets and increased expression, IL-9 may be far more functionally diverse than previously expected, especially on lymphomagenesis. *In vitro*, IL-9 was able to stimulate JAK3-dependent survival of ALK+ anaplastic large-cell lymphoma cells [9] and protect thymic lymphoma cells from dexamethasone-induced apoptosis [26]. *In vivo*, nucleophosmin-ALK expression during transgenic IL-9 expression led to the development of murine plasmacytoma, plasmoblastic/anaplastic lymphoma and precursor T-lymphoblastic lymphoma [27]. IL-9R was markedly overexpressed in diffuse large B-cell lymphoma tissues compared to their counterparts, and it was associated with several adverse prognostic parameters [28]. IL-9 was also expressed in two of six cases of large cell anaplastic lymphoma and in 6 of 13 cases of Hodgkin's disease by northern blot analysis or in situ hybridization [10]. Fischer M *et al*. detected IL-9 in serum samples and found a novel correlation between increased serum IL-9 levels, HL and clinical features [8]. Additionally, Kelleher K *et al*. reported that IL-9 was constitutively expressed in human T-cell leukemia virus-I-transformed human T cells, implying a possible correlation between IL-9 expression and T cell lymphoma/leukemia [29].

ENKTL was previously known as lethal midline granuloma because, macroscopically, the tumor looked like a necrotic granuloma, and the disease showed an aggressive and lethal course [30], [31]. Histologically, ENKTL is characterized by angiocentric and polymorphous lymphoreticular infiltrates, known as polymorphic reticulosis [31], [32]. Previous studies indicated that many cytokines might be responsible for its pathological features. TNF-α induced by Epstein-Barr virus could lead to the observed necrosis [33]. The monokine induced by interferon-γ (IFN-γ), and the IFN-γ-inducible protein-10 play important roles in the pathogenesis of tissue necrosis and vascular damage associated with certain EBV-positive lymphoproliferative processes [4], [5]. Furthermore, Yang *et al*. reported that IL-9 induced by EBV-encoded small RNA acted as an autocrine growth factor for EBV-infected T cells [34]. Furthermore, several studies have mentioned the role of IL-9 in ENKTL. For example, IL-9 was specifically expressed by nasal NK/T-cell lymphoma cell lines, where it acted as an autocrine growth factor, suggesting that the IL-9 signaling pathway may be a new therapeutic target for NK/T-cell lymphoma [6]. However, few studies have focused on the relationship between IL-9 levels and clinical manifestations of ENKTL.

In the present study, we measured serum IL-9 levels in healthy volunteers and patients with ENKTL. We found that IL-9 was fairly low in the healthy controls (average concentration: 0.56 pg/mL), but it showed a relative higher level in patients with ENKTL, with a mean value of 6.48 pg/mL. Age, B symptoms and local lymph node involvement were correlated with high serum IL-9 levels. Due to its role in recruiting non-malignant infiltrating cells, IL-9 makes a crucial contribution to tumor survival, migration and metastasis [35]. Thus, it is easy to explain the correlation between IL-9 and B symptoms and local lymph node involvement. However, it remains unclear why IL-9 levels were also associated with age. According to the ROC curve analyses, 3.49 pg/mL was an optimal cut-off value for distinguishing between poor outcomes. Patients with low IL-9 levels (≤3.49 pg/mL) tended to have higher CR rates (83.3%) than those with high IL-9 levels (>3.49 pg/mL) (57.7%) (*P* = 0.016). Specifically, the median PFS and OS in the low IL-9 level group were clearly longer than those in the high IL-9 level group (PFS: 68.7 months vs. 28.3 months, *P*<0.001; OS: 86 months vs. 42.8 months, *P* = 0.001). Multivariate analysis also indicated that IL-9 was an independent prognostic factor for PFS and OS. All these data confirmed that serum IL-9 was closely correlated with certain clinical features, treatment response and prognosis in ENKTL, implying a significant role for IL-9 in the pathogenesis and development of this disease. Nevertheless, IL-9 can lead to either a favorable or unfavorable clinical prognosis depending on the tumor. Lu Y *et al*. elucidated the role of Th9 cells and the γ chain family member IL-9 in a B16 melanoma mouse model, and they found that Th9-derived IL-9 inhibited tumor progression [36]. This was also supported by Purwar R *et al.*, who indicated that the generation of IL-9-mediated immune responses may have an important role in the treatment of melanoma and other solid tumors [37]. The inconsistent roles of IL-9 in tumors may be partly attributed to different immune effects or single nucleotide polymorphisms [38], [39]. Thus, the exact molecular mechanisms of IL-9 in the pathogenesis and development of ENKTL need to be further clarified.

In conclusion, this is the first study that confirms the close relationship of IL-9 with several clinical features of ENKTL, including age, B symptoms and local lymph node involvement. Serum IL-9, which can be easily measured in clinical practice, may be a significant independent prognostic factor for this disease. These results suggest a role for IL-9 in the pathogenesis of ENKTL and offer new insight into potential therapeutic strategies.
